# Single-incision laparoscopic repair for an arcuate line hernia: a case report

**DOI:** 10.1186/s40792-021-01281-w

**Published:** 2021-08-26

**Authors:** Tomohiko Fukunaga, Toshihiko Kasanami

**Affiliations:** Department of Surgery, Sonobe Hospital, 5-8-7 Misono-cho, Sonobe-cho, Nantan, Kyoto, 622-0002 Japan

**Keywords:** Arcuate line hernia, Arcuate line, Linea arcuata hernia, SILS, IPOM

## Abstract

**Background:**

The arcuate line is the inferior margin of the posterior layer of rectus abdominis sheath. An arcuate line hernia is a parietal interstitial hernia consisting of ascending protrusion of intraperitoneal contents above the arcuate line. Arcuate line hernias are rare, and fewer than 20 cases undergoing surgical repair have been reported. Various surgical approaches were used in previous cases, and there is no consensus regarding the ideal repair method. We report the first case of an arcuate line hernia repaired using single-incision laparoscopic surgery.

**Case presentation:**

The patient was a 78-year-old man who presented with a history of intermittent lower abdominal quadrant pain of more than 2 month’s duration. He had not previously undergone abdominal surgery, but had a history of mycobacterial lung disease and asthma. His vital signs were normal on presentation, and he experienced no vomiting or nausea. On palpation, his abdomen was flat and soft, and no mass was palpable. However, there was slight tenderness in the right lower quadrant. Blood laboratory test results were within normal ranges. Computed tomography revealed small bowel protrusion between the rectus abdominis and the posterior rectus sheath, and an arcuate line hernia was suspected and subsequently confirmed intraoperatively. The patient underwent single-incision laparoscopic repair with the intraperitoneal onlay mesh technique with tacks and with care to avoid the inferior epigastric vessels. The operation time was 30 min, and no intra- or post-operative complications occurred. Surgery relieved his symptoms, with no recurrence within 1 year postoperatively.

**Conclusions:**

Single-incision laparoscopic surgery was performed easily and successfully in this rare patient with arcuate line hernia. Arcuate line hernia should be considered in patients presenting with abdominal symptoms, and single-incision laparoscopic repair should be considered for repair.

## Background

**Fig. 1:**
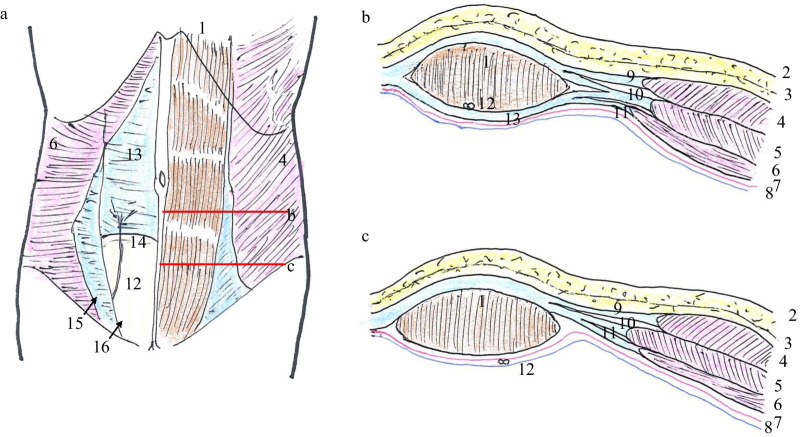
Diagram of the anterior abdominal wall. **a** The right rectus abdominis muscle, external oblique muscle, and internal oblique muscle were resected. **b** Section above the arcuate line. **c** Section below the arcuate line. **1** Rectus abdominis muscle, **2** skin, **3** Scarpa’s fascia, **4** external oblique muscle, **5** internal oblique muscle, **6** transverse abdominis muscle, **7** transversalis fascia, **8** peritoneum, **9** aponeurosis of the external oblique muscle, **10** aponeurosis of the internal oblique muscle, **11** aponeurosis of the transverse abdominis muscle, **12** inferior epigastric vessels, **13** posterior layer of the rectus abdominis, **14** arcuate line, **15** semilunar line

The arcuate line (AL), also called the linea arcuata, linea semicircularis, and the semicircular line of Douglas, marks an anatomical transition point inferior to which all the aponeurotic layers of the abdominal muscles, except the transversalis fascia, pass simultaneously anterior to the rectus abdominis muscle [1, 2]. The arcuate line is the inferior margin of the posterior rectus sheath (PRS). At the caudal side of the AL, the posterior side of the rectus abdominis muscle is covered only by the transversalis fascia and the peritoneum as the areolar tissue layer (Fig. 1). An arcuate line hernia (ALH) is a protrusion of intraperitoneal structures above the PRS, with the hernia orifice between the AL and the rectus abdominis. ALH is generally categorized as an internal or intraparietal hernia, as there is no true abdominal wall defect [3]. This rare type of hernia was first reported by Cappeliez et al. [3] and there have been fewer than 20 reported cases of surgery for ALH to date [3–16]. Surgical management of ALH comprises open or laparoscopic repair, with or without a mesh. We performed single-incision laparoscopic surgery (SILS) for the first time for ALH. The hernia was repaired using the intraperitoneal onlay mesh (IPOM) technique, and the operation was performed safely and easily; therefore, we report SILS as a useful method.

## Case presentation

**Fig. 2:**
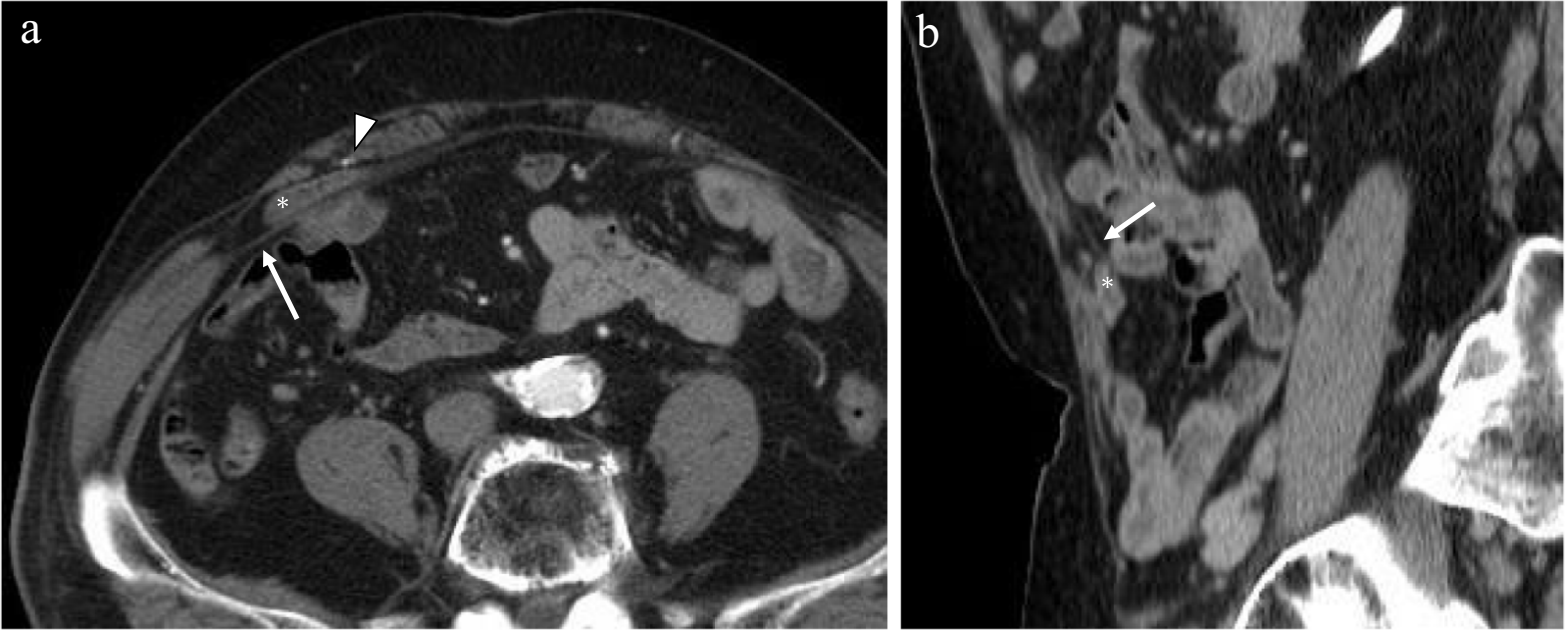
Abdominal contrast-enhanced computed tomography, axial plane (**a**) and sagittal plane (**b**). Protrusion of the small bowel (asterisk) is evident between the rectus muscle and the posterior rectus sheath (arrow). The arrowhead indicates the right inferior epigastric artery

The patient was a 78-year-old man who presented to our hospital with a chief complaint of intermittent right lower abdominal quadrant pain for over 2 months. His medical history included nontuberculous mycobacterial lung disease and asthma, and he had no history of abdominal surgery. He had neither nausea nor vomiting, and his vital signs were normal. His height was 162.2 cm, weight was 72 kg, and body mass index (BMI) was 27.4 kg/m^2^. His abdomen was soft and flat, no parietal mass was palpated, and slight tenderness was present in the right lower quadrant. Blood test results were within normal limits. Abdominal contrast-enhanced computed tomography (CT) revealed protrusion of a small bowel loop between the rectus muscle and the PRS (Fig. 2); ALH was suspected. Laparoscopic surgery was planned, and SILS was chosen to minimize the invasiveness.

**Fig. 3:**
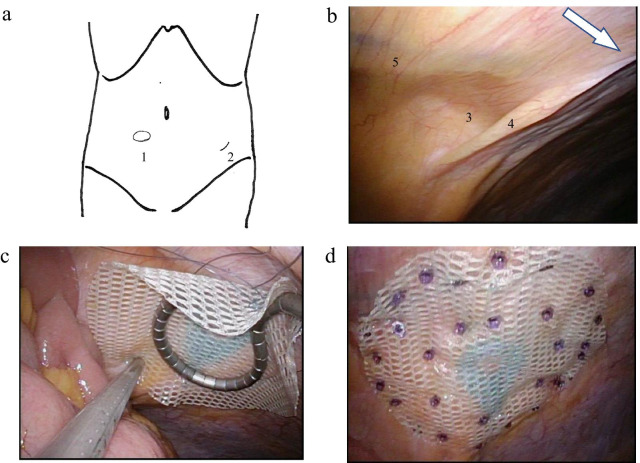
Operative findings: **a** location of the hernia (**1**) and skin incision (**2**). **b** The arrow indicates the cranial side. The hernia (**3**) is above the arcuate line (**4**) and lies along the inferior epigastric vessels (**5**). **c** The first tack to fix the mesh involved using the Diamond-Flex® circular retractor to stabilize the mesh position. **d** The mesh is secured using the double-crown method

With the patient in the supine position under general anesthesia, a 2-cm-long muscle-splitting incision was made at contralateral McBurney’s point. A Lap Protector™ (Hakko, Nagano, Japan) was attached to the wound, and an EZaccess™ (Hakko) with three inserted 5-mm trocars was added (Fig. 3a). Laparoscopically, the ALH was easily detected, and there were no hernia contents (Fig. 3b). We used the IPOM technique for its simplicity and repair reliability. A 9-cm-diameter Symbotex™ (Medtronic, Minneapolis, Minnesota, USA) composite mesh was positioned so that the AL was pressed against the rectus abdominis muscle, and the mesh was fixed using AbsorbaTack™ (Medtronic) tacks while percutaneously compressing from the opposite side. The first few tacks were fixed using a Diamond-Flex® circular retractor (BD, Franklin Lakes, New Jersey, USA) to stabilize the mesh position, and the final 30 tacks were fixed using the double-crown technique (Fig. 3c and d). We fixed the mesh carefully to avoid damaging the inferior epigastric vessels. Seprafilm® (Sanofi, Paris, France) was placed under the incision to prevent adhesions, and the wound was sutured in layers. The operation time was 30 min. The patient’s postoperative course was uneventful, and he was discharged on the third post-operative day. His symptoms resolved after surgery, and no recurrence has been noted 1 year since the surgery.

## Discussion

In the supraumbilical and part of the infraumbilical abdominal wall, the rectus abdominis muscle is covered on the dorsal side by the PRS, a plate of aponeurotic tissue that is formed by the fascia transversalis and posterior lamina of the internal oblique muscle. Somewhere between the umbilicus and pubic bone, the posterior lamina of the internal oblique muscle joins the anterior lamina on the ventral side of the rectus muscle, leaving only the transversus fascia on the dorsal side as areolar tissue [17]. This level is called the AL. In other words, the AL can be described as the inferior margin of the PRS. The level of the AL varies. According to a cadaveric study by Loukas et al. the AL was located a mean of 2.1 ± 2.3 cm superior to the level of the anterior superior iliac spines [2]. It is thought that ALHs form by the folding of the peritoneum and the transversus fascia between the dorsal transversus abdominis and the PRS. ALH is classified as an internal hernia because there is no true defect in the abdominal wall [3]. The ALH orifice is wide, and Montgomery et al. described it as ‘the top of a mitten’ [12]. Therefore, abdominal organs are able to move in and out of the hernia easily, which may have caused the intermittent symptoms described by our patient.

**Fig. 4:**
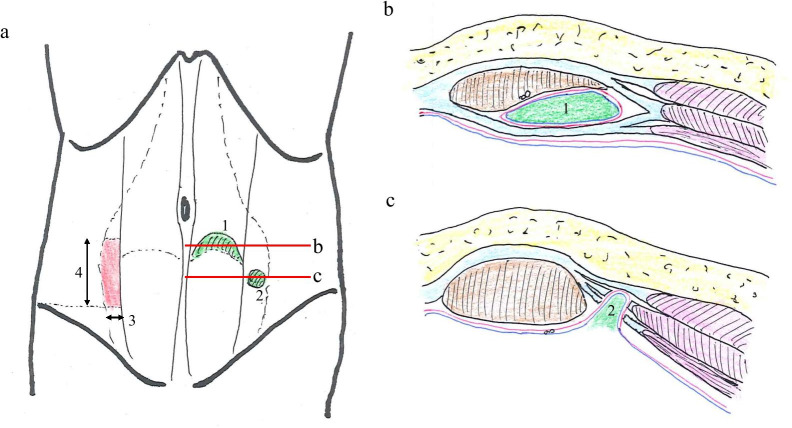
Anatomical location of arcuate line hernia (**1**) and Spigelian hernia (**2**). (**3**) Spigelian aponeurosis, between the semilunar line (muscular–aponeurosis transition of the transverse abdominis muscle) and the lateral edge of the rectus abdominis. (**4**) Spigelian hernia belt

In some cases, the preoperative diagnosis of ALH was mistaken for Spigelian hernia [3, 12]. Spigelian hernias are located at the level of the semilunar line where the fasciae of the oblique and transversus muscles begin to split into separate layers of the abdominal musculature. Spigelian hernias account for 1%–2% of abdominal wall hernias [18]. The majority occur within the ‘Spigelian hernia belt’, which is the 6-cm area of the Spigelian aponeurosis that lies cephalad to the interspinal plane [19]. Spigelian hernias often have a narrow fascial defect and, therefore, have an increased risk of incarceration and strangulation [20]. Both ALH and Spigelian hernia occur at a similar level, but the position of the hernia portal is slightly lateral in Spigelian hernias compared with ALH (Fig. 4). Knowledge of both hernias is the key to diagnosis.

The prevalence of ALH is unclear, and the underlying reason is that most ALHs are asymptomatic and remain unclassified, and the diagnosis is often incidental or could be misclassified as another abdominal hernia. To investigate the incidence of ALH, two retrospective large cohort studies using CT have been reported [17, 21]. Courier et al. retrospectively analyzed a continuous series of 315 unselected patients and classified AL abnormalities. A delineation of the AL with minimal bulging of intraperitoneal fat was classified as grade 1 (G1). Grade 2 (G2) herniation was defined as a minimal but substantial true herniation of fat and/or intestinal loops under the AL. Grade 3 (G3) was defined as a clear prominent herniation of abdominal structures (omental fat and/or bowel); G2 and G3 were defined as ALH. In the series, AL abnormality (G1, G2, and G3) was found in 8.57% of the patients, and actual ALH (G2, G3) was found in 1.62% of the patients. The prevalence of AL abnormality (G1, G2, and G3) is higher in men, with a reported M:F ratio of 12.5:1 [21]. Bloemen et al. [17] retrospectively analyzed 415 patients who presented to the emergency department for surgical consultation with abdominal complaints and who underwent CT but did not have a definitive diagnosis. ALH was classified according to the definition of Courier et al. In the series, AL abnormality was found in 11.3% of the patients, and actual ALH was found in 3.4% of the patients. The rate of AL abnormality was equally divided among men and women. Patients with ALH were found to have a significantly higher BMI when compared with patients without ALH. Diabetes mellitus and the presence of an aneurysm of the abdominal aorta were also seen more often in patients with ALH. Among patients with ALH, correlation with clinical complaints was found in half of the patients [17]. The results of these two studies suggest that it is important to suspect ALH in patients with abdominal complaints.

There have been 19 reported cases of surgery for AL (Table 1) [3–16]. Unexpectedly, the ratio of men to women was 8:11; slightly more women than men. Three cases occurred after abdominal surgery and were diagnosed as incisional hernias, but were diagnosed as ALH intraoperatively [4, 15, 16]. As previously mentioned, ALH is considered less likely to cause symptoms because of the wide hernia orifice; however, there have been cases of emergency surgery owing to incarceration [6, 9, 13]. Although ALH is a rare and little-known disease, these cases suggest that surgeons should be aware of the condition.

**Table 1:** Summarized data from published reports of arcuate line hernia

Authors	Year	Age/sex	Symptoms	History of abdominal surgery	Examinations	Preoperative diagnosis	ALH side	Hernia content	Incarceration	Other Hernia comorbidities	Surgical approach	Repair method
Cappeliez et al.	2003	73 M	Right flank pain	N/A	CT	ALH	Bilateral	Small bowel	Not incarcerated	No	Laparoscopy	Mesh repair
von Meyenfeldt et al. (case 1)	2009	53 M	Bulge in the left paramedian abdomen	No	US	Spigelian hernia	Left	N/A	Not incarcerated	No	Open laparotomy	Mesh repair
von Meyenfeldt et al. (case 2)	2009	41 M	Bulge in the left paramedian abdomen	Non-mesh repair of a left inguinal hernia	CT	Spigelian hernia	Left	Small bowel	Not incarcerated	No	Diagnostic laparoscopy to open laparotomy	Mesh repair
Abasbassi et al.	2011	53 F	Left lower abdominal quadrant pain	No	CT	ALH	Left	Sigmoid colon	N/A	No	Laparoscopy	Transected posterior rectus sheath
Montgomery et al. (case 1)	2013	49 F	Abdominal pain on the right side	No	CT	Umbilical hernia with two unusual internal herniations	Bilateral	Small bowel	Not incarcerated	Umbilical hernia	Laparoscopy	TAPP
Montgomery et al. (case 2)	2013	79 M	Umbilical pain	Left inguinal herniorrhaphy and cholecystectomy	N/A	N/A	Right	N/A	Not incarcerated	Umbilical hernia	Laparoscopy	TAPP
Montgomery et al. (case 3)	2013	78 M	Right inguinal pain	Appendectomy	N/A	Bilateral inguinal hernia	Left	N/A	Not incarcerated	Bilateral inguinal hernia	Laparoscopy	Spiral tacks
Messaoudi et al. (case 1)	2014	57 F	Intermittent abdominal pain	No	CT	ALH	Left	Small bowel	Not incarcerated	No	Laparoscopy	IPOM
Messaoudi et al. (case 2)	2014	39 F	Prominent incision	Anterior lumbar fusion of the L5–S1 segments	N/A	Incisional hernia	Left	N/A	Not incarcerated	No	Laparoscopy	IPOM
Verlynde et al.	2016	56 F	Right lower abdominal quadrant pain	N/A	US and CT	ALH	Right	Parietal peritoneal lipomatous appendage and lipomatous epiploic appendage of the sigmoid colon	Incarcerated	No	Laparoscopy	TAPP
Vincelli et al.	2017	46 F	Abdominal pain and vomiting	Cesarean section with Pfannenstiel incision	CT	Incisional hernia	N/A	small bowel	N/A	No	Open laparotomy	Direct suture
Weimer et al.	2017	51 F	Abdominal pain and nausea	N/A	CT	ALH	Bilateral	Small bowel	Not incarcerated	No	Robot	TAPP
Hugot et al.	2017	64 F	Right flank pain	Laparoscopic cholecystectomy	CT	ALH	Right	Omental fat	Incarcerated	No	Laparoscopy	TAPP
Bloemen et al.	2018	79 M	N/A	N/A	CT	Obstructive ileus with suspected incarcerated ventral hernia	Left	N/A	Incarcerated	N/A	Laparoscopy	N/A
McCulloch et al.	2018	68 F	Sudden-onset fever, nausea, vomiting, and abdominal discomfort	Incisional hernia repair after emergency laparotomy	CT	Incisional hernia	N/A	Small bowel and colon	Incarcerated	No	Open laparotomy	Mesh
Coulier et al.	2019	59 F	Alternating episodes of diarrhea and constipation	N/A	CT	ALH	Bilateral	Small bowel and transverse colon	N/A	N/A	N/A	N/A
Berney et al.	2019	59 F	Infraumbilical lump	N/A	CT	Midline ventral hernia	Bilateral	Small bowel	Not incarcerated	Midline ventral hernia	Hybrid laparoscopic and open surgery	IPOM
Kollias et al.	2021	46 M	Left-sided abdominal pain	Mesh repair of a left inguinal hernia	CT	ALH	Left	Small bowel	Not incarcerated	No	Laparoscopy	eTEP
Our case	2021	78 M	Right lower abdominal quadrant pain	No	CT	ALH	Right	Small bowel	Not incarcerated	No	Single-incision Laparoscopic surgery	IPOM

*ALH* arcuate line hernia, *M* male, *F* female, *N/A* not available, *CT* computed tomography, *US* ultrasonography, *TAPP* transabdominal preperitoneal repair, *eTEP* extended-view totally extraperitoneal repair, *IPOM* intraperitoneal onlay mesh

Laparoscopic surgery was performed in several cases, and is recommended for several reasons. The diagnostic properties of laparoscopy are superior, making it possible to diagnose concomitant hernias that could be addressed during the same operation, and for excluding other diagnoses, especially in emergency surgery. Laparoscopy also enables inspecting the contents of the hernia sac. It is also considered possible that asymptomatic ALH may be found during laparoscopic surgery for other diseases. Whether asymptomatic ALH needs to be repaired is a matter of debate. In reported cases, the methods of hernia repair comprised direct suture, trans-abdominal preperitoneal repair (TAPP), and extended-view totally extraperitoneal repair (eTEP). There have been no reports of recurrence. It is difficult to determine the best method because each technique has advantages and disadvantages. It is best to use the method that the surgeon is comfortable with. In any method, care must be taken to avoid damaging the inferior epigastric vessels that branch from the external iliac vessels and cross the AL into the rectus abdominis. In our case, we performed SILS using the IPOM technique. The procedure was relatively simple, and we were able to close the hernia orifice safely and securely. The advantages of SILS are reduced associated morbidity, namely wound infection, pain, bleeding, visceral injury, and port-site herniation [22]. In light of the above, SILS should be considered an option for ALH.

## Conclusion

We reported the first case of SILS for ALH. Although ALH is a rare disease, its possibility should be considered in patients with abdominal complaints. Laparoscopy is recommended for symptomatic ALH, and SILS should be considered.

## Data Availability

All data generated or analyzed during this study are included in this published article.

## Abbreviations

ALH: Arcuate line hernia
AL: Arcuate line
PRS: Posterior rectus sheath
SILS: Single-incision laparoscopic surgery
IPOM: Intraperitoneal onlay mesh
BMI: Body mass index
CT: Computed tomography
